# Roger on Infectious Diseases

**Published:** 1904-05

**Authors:** 


### Books and Magazines

ROGER ON INFECTIOUS DISEASES.—Their Etiology, Diagnosis and Treatment. By G. H. Roger, Professor Extraordinary in the Faculty of Medicine of Paris, etc.; translated by M. S. Gabriel. M. D., New York. In one octavo volume of 864 pages, with 43 illustrations. Cloth, $5.75 net. Lea Brothers & Co., Philadelphia and New York. 1903.

Professor Roger has had opportunities for the study of infectious diseases which rarely fall to the lot of any man. In the hospitals under his charge are received all cases of contagious diseases which occur in Paris, and he has personally attended more than 10,000 patients during a period of five years. The effect and purpose of this work is to harmonize any seeming antagonism between experimental researches and clinical observation and to reduce the theories of infection and immunity to a basis of practical utility.

